# Role of the Retinoblastoma protein, Rb, during adult neurogenesis in the olfactory bulb

**DOI:** 10.1038/srep20230

**Published:** 2016-02-05

**Authors:** Rayan Naser, Renaud Vandenbosch, Saad Omais, Dayana Hayek, Carine Jaafar, Sawsan Al Lafi, Afaf Saliba, Maarouf Baghdadi, Larissa Skaf, Noël Ghanem

**Affiliations:** 1 Department of Biology, American University of Beirut, Lebanon; 2 Department of Cellular and Molecular Medicine, University of Ottawa, Canada

## Abstract

Adult neural stem cells (aNSCs) are relatively quiescent populations that give rise to distinct neuronal subtypes throughout life, yet, at a very low rate and restricted differentiation potential. Thus, identifying the molecular mechanisms that control their cellular expansion is critical for regeneration after brain injury. Loss of the Retinoblastoma protein, Rb, leads to several defects in cell cycle as well as neuronal differentiation and migration during brain development. Here, we investigated the role of Rb during adult neurogenesis in the olfactory bulb (OB) by inducing its temporal deletion in aNSCs and progenitors. Loss of Rb was associated with increased proliferation of adult progenitors in the subventricular zone (SVZ) and the rostral migratory stream (RMS) but did not alter self-renewal of aNSCs or neuroblasts subsequent migration and terminal differentiation. Hence, one month after their birth, Rb-null neuroblasts were able to differentiate into distinct subtypes of GABAergic OB interneurons but were gradually lost after 3 months. Similarly, Rb controlled aNSCs/progenitors proliferation *in vitro* without affecting their differentiation capacity. This enhanced SVZ/OB neurogenesis associated with loss of Rb was only transient and negatively affected by increased apoptosis indicating a critical requirement for Rb in the long-term survival of adult-born OB interneurons.

Adult neurogenesis is a dynamic developmental process by which new and functional neurons are generated from adult neural stem cells (aNSCs) in the mammalian brain throughout life^1^,^2^,^3^. Much progress has been made to understand the properties of aNSCs and the supporting role attributed to the local environment or niche as well as the distinct steps of adult neurogenesis in rodents^4^,^5^,^6^ and humans^7^,^8^,^9^,^10^.

In the adult brain, aNSCs reside in the subventricular zone (SVZ) lining the lateral ventricles where they produce neuroblasts that migrate along the rostral migratory stream (RMS) to the olfactory bulb (OB) and generate inhibitory neurons, and, the subgranular zone (SGZ) in the hippocampus (Hi) that gives rise to new granule cells in the dentate gyrus^1^,^11^,^12^,^13^,^14^,^15^. The adult SVZ harbors four distinct cell types: 1) the ependymal cells or type E lining the lateral ventricles (LV), 2) the multipotent astrocyte-like stem cells or type B which are relatively quiescent and self-renewing, 3) the transient-amplifying cells or type C that are derived from type B cells and proliferate rapidly to generate 4) immature neuroblasts or type A which ultimately differentiate into neurons^12^,^13^,^16^. Adult neurogenesis contributes to brain homeostasis and plasticity as well as brain regenerative capacity under normal physiological conditions and after brain injury. Hence, controlled expansion of aNSCs/progenitors followed by their targeted differentiation into desired lineages may lead to important therapeutic interventions. However, the restricted number of these cells and their low regenerative rate are still major obstacles facing this aim.

Previous studies have shown that cell cycle genes are key regulators of cell cycle progression and control the size of different neural populations in the brain in coordination with cell fate markers and differentiation genes^17^,^18^,^19^. For instance, the tumor suppressor gene, Rb, regulates proliferation and migration of neuronal progenitors during brain development^20^,^21^,^22^,^23^. Moreover, we have shown that the Rb/E2F pathway modulates neuronal differentiation through direct regulation of *Dlx* homeobox genes during late embryogenesis; hence, telencephalic-specific deletion of Rb results in abnormal progenitor differentiation in the SVZ and migration along the RMS and inside the OB^24^. Here, we have investigated the function of Rb in adult neurogenesis in the OB by inducing its temporal deletion specifically in aNSCs and progenitors using Nestin-CreERT2-Rosa26-YFP and retroviral-mediated Cre delivery. We report that loss of Rb enhances proliferation of adult progenitors found in the SVZ and the RMS but does not affect self-renewal of aNSCs. Moreover, we show that Rb-null adult neuroblasts exit properly the cell cycle and differentiate into mature neurons both *in vivo* and *in vitro* similar to Rb+/− littermate controls. Thus, unlike during development, these neuroblasts do not display any obvious migration or differentiation defects and give rise to distinct subtypes of GABAergic OB interneurons one month after their birth in the SVZ. However, this enhanced neurogenesis in the SVZ/OB associated with loss of Rb was only transient and negatively affected by increased apoptosis of Rb-null adult-born interneurons after longer survival periods.

## Results

### Temporal deletion of Rb in aNSCs and progenitors

**Figure 1:**
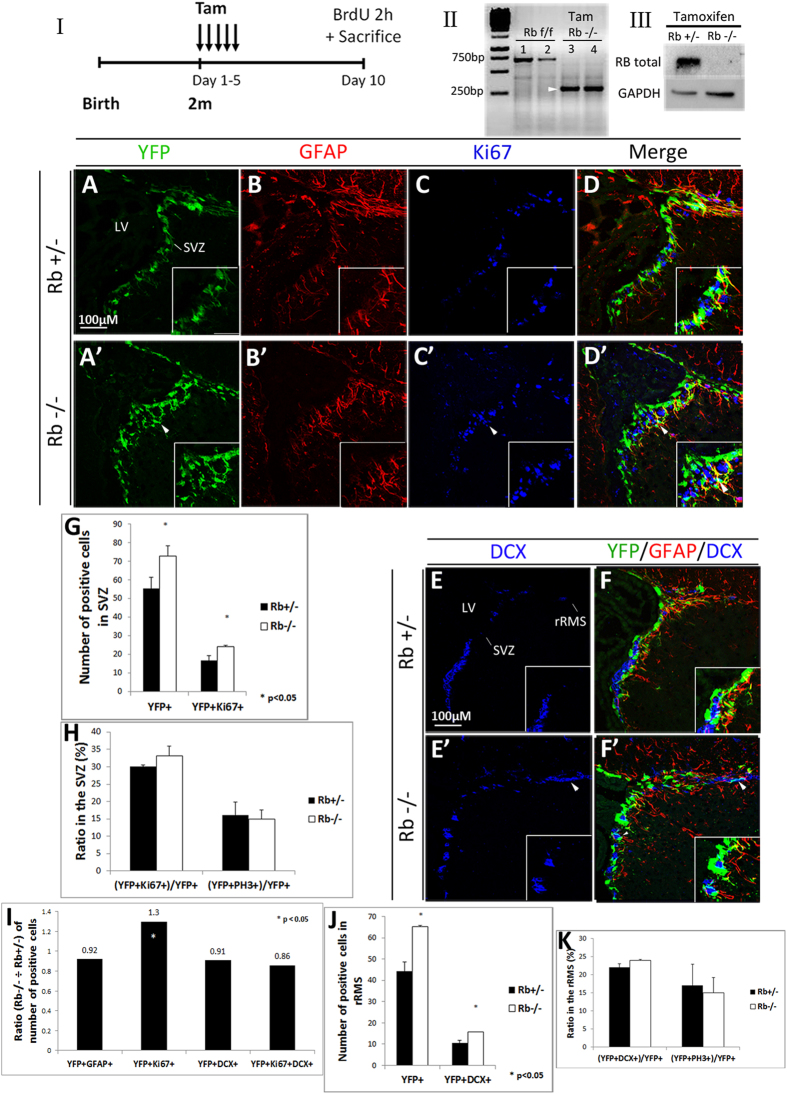
Induced temporal deletion of Rb in aNSCs and progenitors in the SVZ. (**I**) Experimental design for the temporal deletion of Rb: animals received 5 tamoxifen (Tam) injections (1 injection per day) at 2 months (2m) of age and a single BrdU injection 2 h prior to sacrifice on day 10. (**II**) PCR analysis of gDNA extracted from Rb^flox/flox^ neurospheres (1, 2) and Rb−/− green fluorescent neurospheres (3, 4): arrow point to a 320 bp band corresponding to the Rb cleaved allele. (**III**) Western blot analysis of protein extracts from Rb+/− and Rb−/− green fluorescent neurospheres showing loss of Rb protein. (**A**–**D**′) Triple immunohistochemistry performed on sagittal sections against YFP, GFAP and Ki67 in the SVZ in Rb+/− (**A**–**D**) and Rb−/− (**A′**–**D′**) mice (n = 3Ct and 3Mut). (**E**–**F′**) Triple staining with anti-YFP, anti-GFAP and anti-DCX in Rb+/− (**E**,**F**) and Rb−/− (**E′**,**F′**) mice (n = 3Ct and 3Mut). Arrowheads in (**A′**–**F′**) depict increased numbers of stained cells in Rb−/− compared with Rb+/− mice. Insets in (**A**–**F′**) are higher magnifications of a selected region in the medial SVZ. (**G**,**H**) quantifications of YFP+ and YFP+Ki67+ cells in the SVZ and the ratios of double positive cells over total YFP+ cells, respectively. (**I**) shows the ratios (Mut ÷ Ct) of YFP+ cells co-labeled with distinct cell markers. (**J**,**K**) quantifications of YFP+ and YFP+DCX+ cells in the rRMS and the ratios of double positive cells over total YFP+ cells, respectively. Error bars represent SD of measurements from at least n = 3 per genotype and asterisks indicate a statistically significant difference between genotypes using independent samples t-tests. Scale bar = 100 uM. f; flox, LV; lateral ventricle, rRMS; rostral part of the rostral migratory stream, SVZ; subventricular zone.

Considering the central role played by the Rb/E2F pathway in embryonic neurogenesis^24^, we investigated the role of Rb in the adult mouse brain by inducing its temporal deletion specifically in aNSCs and progenitors. Hence, we crossed Nestin-CreERT2; Rosa26^YFP/YFP^ transgenic mice^25^ with Rb^flox/flox^ mice^26^ to generate Rb control animals (Nestin-CreERT2; Rosa26^YFP/+^; Rb^flox/+^) and Rb mutant animals (Nestin-CreERT2; Rosa26^YFP/+^; Rb^flox/flox^), thereafter referred to as Rb+/− and Rb−/− after tamoxifen treatment, respectively. 8-week-old mice received 5 tamoxifen injections over 5 days (5 d) and were sacrificed 5 d, 28 d, 60 d, and 120 d, later. A single BrdU pulse was administered to all animals 2 h prior to sacrifice (Fig. 1I; refer to methods for detail). First, we used genomic DNA and protein extracts from YFP-expressing neurospheres derived from cultured SVZ tissue and confirmed by PCR that the Cre recombination was successful *in vivo* (Fig. 1II) and, by Western blot analysis, that the Rb protein was deleted following tamoxifen treatment (Fig. 1III). Next, we assessed YFP expression by immunostaining 5 d post-deletion of Rb and detected green/recombinant cells in the dorsal, medial and ventral regions of the SVZ lining the LV as well as in dispersed cells inside the dentate gyrus in both Rb+/− and Rb−/− animals but not in vehicle-treated animals (Fig. 1A,A′ and data not shown). The number of YFP+ cells found in the SVZ and the intensity of the YFP fluorescent signal were remarkably higher in Rb−/− animals compared with controls (Fig. 1A,A′; arrowhead, 1G:YFP+: 54.5 ± 5.96 in Rb+/− vs 73 ± 5.4 in Rb−/−, mean ± S.D., p = 0.037). This was also true for YFP+ cells co-expressing Ki67, a nuclear protein expressed in actively dividing cells, mainly type C cells in the adult SVZ, and, for a short period of time (<1 day) in new-born post-mitotic neurons^27^ (Fig. 1C–D′; arrowheads, 1G: YFP+;Ki67+: 15.6 ± 2.7 in Rb+/− vs 24.2 ± 0.5 Rb−/−, mean ± S.D., p = 0.033). Moreover, the proliferative index represented by the ratio of [YFP+Ki67+] cells over the total number of YFP+ cells and the mitotic index [(YFP+PH3+)/YFP+), PH3: phospho-Histone H3, M phase marker] were similar between genotypes suggesting the presence of increased cell proliferation and subsequent division rather than an enhanced rate of cell division (speed of cell cycle) (Fig. 1H). We then examined the percentages of type B and A cells found among the recombinant progeny by labeling for the Glial Fibrillary Acidic Protein (GFAP), an astrocyte-specific marker that identifies also astrocyte-like NSCs (type B) and Doublecortin (DCX), a microtubule-binding protein expressed in late progenitors and neuroblasts (type A) but downregulated when neurons become mature^28^. Triple labeling against YFP/GFAP/Ki67, YFP/GFAP/DCX and YFP/DCX/Ki67 revealed a significant increase in the number of YFP+ cells co-expressing Ki67 in the SVZ in Rb−/− compared with Rb+/− animals as indicated by the ratio of these double positive cells in the mutant versus control animals (Fig. 1A–D′: r = 1.3, p = 0.021). This increase in cell proliferation primarily affected the pool of progenitors and not aNSCs since the number of [YFP+GFAP+Ki67] cells did not change between genotypes nor the ratio of YFP+ cells co-expressing GFAP (Fig. 1A–D′; ratio = 0.92 and data not shown). There was no significant difference in the size of other SVZ populations between genotypes as shown by the similar ratios of YFP+ cells co-expressing DCX (Fig. 1E–F′; r = 0.91) or both DCX and Ki67 (Fig. 1I; r = 0.86 and data not shown). These results indicate that loss of Rb primarily leads to an expansion in the pool of proliferating progenitors but does not seem to affect other cell types found in the SVZ. Next, we investigated further how Rb controls adult neurogenesis in the SVZ, RMS and OB by studying the phenotype of Rb−/− mice 28 d post-deletion of Rb.

### Rb regulates proliferation of adult progenitors but not aNSCs in the SVZ at 28 d post-Rb deletion

**Figure 2:**
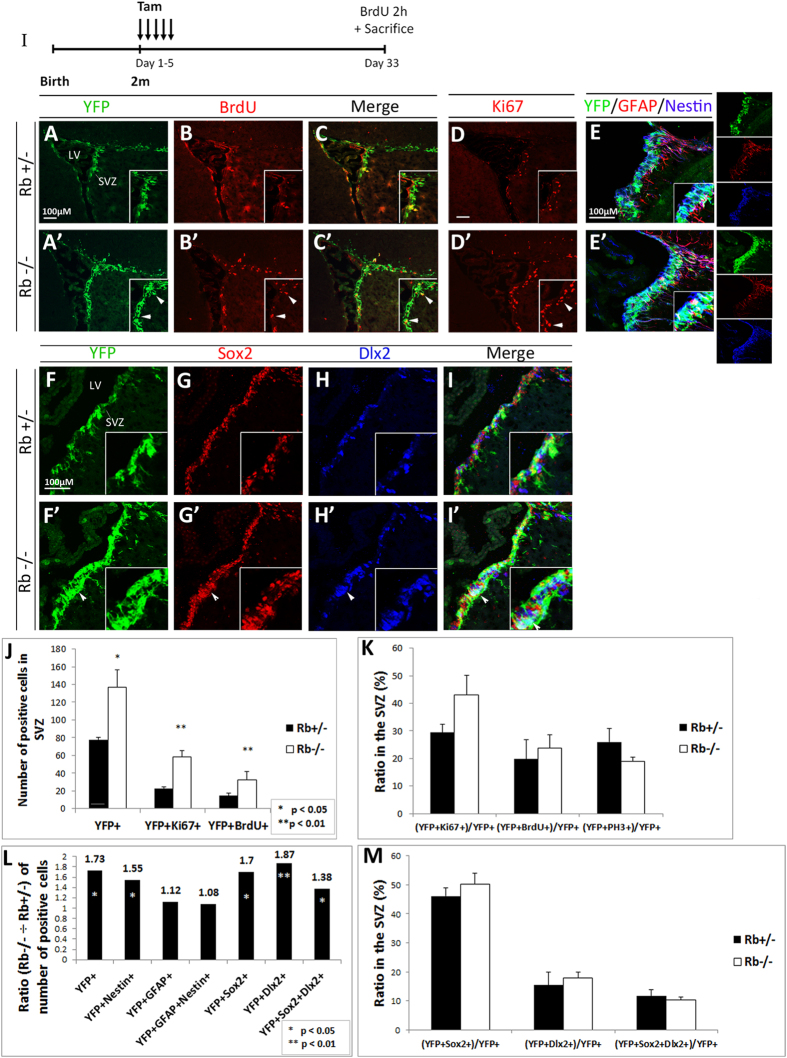
Rb-null mice display enhanced progenitor proliferation in the adult SVZ. (**I**) Experimental design for the temporal deletion of Rb. Animals were sacrificed 28 d post-tamoxifen treatment. (**A**–**C′**) Double immunolabeling performed on sagittal sections with anti-YFP and anti-BrdU, and (**D**,**D′**) anti-Ki67 showing increased cell proliferation in the SVZ (arrowheads in insets **A′**–**D′**) (n = 5Ct and 5Mut). (**E**,**E′**) Triple staining with antibodies against YFP, GFAP and Nestin showing an increase in YFP+ and/or Nestin+ cells in the mutant SVZ compared to controls (n = 3Ct and 3Mut). (**F**–**I′**) Triple labeling with anti-YFP, anti-Sox2 and anti-Dlx2 in the SVZ in Rb+/− (**F**–**I**) and Rb−/− (**F′**–**I′**) mice highlighting the increase in distinct progenitor populations in the absence of Rb (arrowheads in **F′**–**I′**) (n = 3Ct and 3Mut). Insets in (**A**–**I′**) are higher magnifications of selected regions in the medial SVZ. (**J**,**K**) quantifications of YFP+, Ki67+ and/or BrdU+ cells in the SVZ, and the ratios of double positive cells over total YFP+ cells in Rb+/− versus Rb−/− mice, respectively. Note that the proliferation index, [(YFP+BrdU+) or (YFP+Ki67+) ÷ total YFP+], did not change between genotypes. (**L**) depicts the ratios (Rb−/− ÷ Rb+/−) of the different cell populations found in the SVZ; note the increase in YFP+ progenitors co-expressing Nestin, Sox2 and Dlx2. (**M**) graph showing the ratios of YFP-positive cells co-expressing Sox2, Dlx2 or both over total YFP+ cells in the SVZ. Error bars represent SD of measurements from at least n = 3 per genotype and asterisks indicate a statistically significant difference between genotypes using independent samples t-tests. Scale bar = 100 uM.

To further investigate the role of Rb in adult neurogenesis, we examined cell proliferation in the SVZ 28 d post-Rb deletion (Fig. 2I). Consistent with our previous results, we detected a significant increase in the numbers of YFP+ cells co-expressing Ki67 and BrdU in the SVZ in Rb−/− mice compared with Rb+/− mice (Fig. 2A–D′; arrowheads in insets, 2J: YFP+ cells: Rb+/−: 77.6 ± 2.7 vs Rb−/−: 136.7 ± 19.5, r = 1.76, p = 0.032, Ki67+;YFP+ cells: Rb+/−: 22.7 ± 1.8 vs Rb−/− 58.7 ± 6.5, r = 2.5, p = 0.007 and YFP+;BrdU+ cells: Rb+/−: 11.7 ± 2.41 vs Rb−/− 25.77 ± 7.11, r =  2.2, p = 0.005, mean ± S.D.). Also, the proliferative indices [(YFP+BrdU+)/YFP+ and (YFP+Ki67+)/YFP+] and the mitotic index [(YFP+PH3+)/YFP+] were similar between genotypes suggesting the presence of increased cell proliferation along with a proportional increase in cell division (Fig. 2K). In order to determine whether this increase in proliferation affected the number of NSCs and/or progenitors, we co-stained for YFP/GFAP/Nestin and found that 75–85% of Nestin+ cells co-expressed YFP in the SVZ in both genotypes, however, the ratio [Rb−/− ÷ Rb+/−] of [YFP+Nestin+; NSCs and progenitors] cells was significantly increased in the absence of Rb (Fig. 2E–E′: r = 1.55 and data not shown). We did not detect any difference in the ratios of [YFP+GFAP+; NSCs/mature astrocytes] and [YFP+GFAP+Nestin+; NSCs] cells between genotypes (Fig. 2L: r = 1.12 and 1.08, respectively), thus indicating that Rb does not seem to regulate the rate of division of aNSCs. To further confirm this observation, we performed a long-term labeling experiment and used BrdU to mark slow-proliferating cells in the SVZ including aNSCs but not fast-dividing progenitors. Animals received 5 consecutive BrdU injections over 10 h on the third day following their last tamoxifen injection and were sacrificed 28 d later. Results showed no difference in BrdU incorporation in the SVZ in Rb−/− compared with Rb+/− and vehicle-treated animals, indicating no change in the self-renewal capacity of Rb-null aNSCs (data not shown). Next, we assessed how loss of Rb impacts the size of progenitor populations by staining for Sox2, a marker of early precursors and type C progenitors, and Dlx2, a marker of mid-to-late progenitors and some immature neuroblasts (type A). We found that these progenitor populations were proportionally expanded in the mutant SVZ as shown by the ratios [Rb−/− ÷ Rb+/−] of YFP+ cells co-expressing Sox2 (r = 1.7), Dlx2 (r = 1.87) and both markers (r = 1.38) (Fig. 2L), and the fact that the percentages of these populations with respect to the total population of YFP+ cells were unaffected between genotypes (Fig. 2F–I′; arrowheads, 2M). Altogether, our results indicate that Rb specifically regulates progenitor proliferation in the SVZ without affecting the rate of division of aNSCs and this is consistent with its role during embryonic development^22^.

### Rb-null progenitors migrate to the OB and differentiate into adult-born neurons similar to Rb+/− cells one month post-Rb deletion

**Figure 3:**
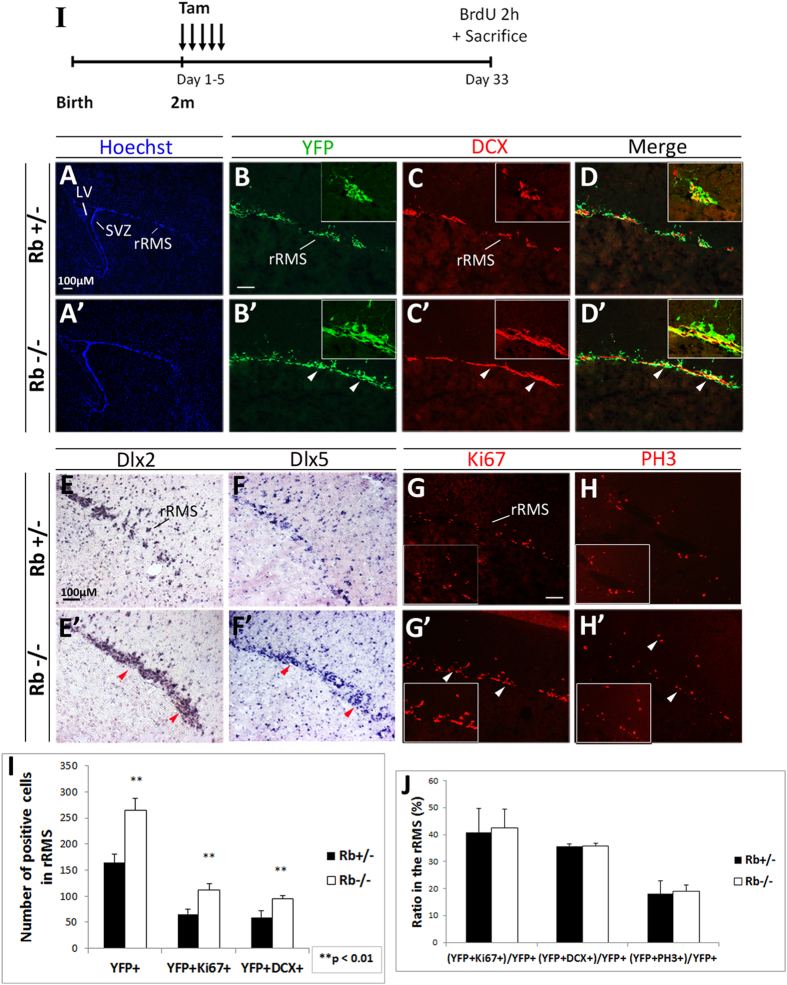
Rb-null neuroblasts migrate along the RMS to the OB similar to Rb+/− control cells. (**I**) Experimental design for the temporal deletion of Rb. (**A**–**D′**) Immunolabeling with Hoechst, YFP and DCX on sagittal sections showing an increase in the number of neuroblasts migrating in the rostral part of RMS (rRMS) in Rb−/− versus Rb+/− (arrowheads in **B′**–**D′**) (n = 3Ct and 3Mut). This increase was confirmed by *in situ* hybridization using RNA labeled anti-sense probes for two neuronal differentiation markers; *Dlx2* (**E**,**E′**) and *Dlx5* (**F**,**F′**) (red arrowheads in **E′**,**F′**) (n = 3Ct and 3Mut). The enhanced neuroblast migration was associated with higher proliferation in the rRMS and normal cell cycle exit as shown by Ki67 (**G**,**G′**; arrowheads) and PH3 staining (**H**,**H′**; arrowheads), respectively. (**I**) Quantification of YFP+ cells co-labeled with DCX or Ki67 in the rRMS. (**J**) Graph showing the proliferation, migration and mitotic indices [double positive cells ÷ total YFP cells] in both genotypes. Error bars represent SD of measurements from n = 3 per genotype and asterisks indicate a statistically significant difference between genotypes using independent samples t-tests. Scale bar = 100uM.

Owing to the enhanced progenitor proliferation observed in the Rb−/− SVZ, we examined whether neuroblast migration is affected in the absence of Rb. Between 2–7 days after their birth in the SVZ, immature neuroblasts start migrating along the RMS to the OB while undergoing their last round(s) of cell division^29^,^30^. We detected a significant increase in the number of DCX+ neuroblasts found in the rostral part of the mutant RMS (rRMS) 5 d after loss of Rb (Fig. 1E–E′; arrowhead) and more so, at 28 d post-treatment (Fig. 3C,C′; arrowheads), including more DCX+ neuroblasts co-expressing YFP (Figs 1F–F′,J and 3B–D′; arrowheads, 3I; [DCX+YFP+] cells: 58.64 ± 13.95 in Rb+/− vs 94.9 ± 5.67 in Rb−/−, r = 1.62, p = 0.008, mean ± S.D.). We further confirmed this by labeling for *Dlx2* and *Dlx5*, two differentiation markers, both of which also showed expanded expression in the rRMS (Fig. 3E–F′; arrowheads) as well as in the medial and caudal parts of the RMS (data not shown). Remarkably, the expansion in the population of neuroblasts throughout the RMS was associated with enhanced cell proliferation as assessed by Ki67 staining (Fig. 3G–G′: [Ki67+YFP+] cells: 66.0 ± 8.7 in Rb+/− vs 111.7 ± 12.0 in Rb−/−, r = 1.68, p = 0.008, mean ± S.D.) and BrdU staining (data not shown), and a proportional increase in mitotic cell division in the same region as indicated by the number of PH3+ cells including [YFP+PH3+] cells (Fig. 3H–H′ and data not shown). This was further confirmed by unaffected indices of cell proliferation, division and migration between genotypes: [(YFP+Ki67+)/YFP+], [(YFP+PH3+)/YFP+], [(YFP+DCX+)/YFP+], respectively (Figs 1K and 3J). Finally, the percentage of [YFP+DCX+Ki67+] cells was also very close between genotypes at all ages examined, thus excluding the presence of any ectopic neuroblast proliferation in the SVZ or the RMS (Fig. 1I; r = 0.86 and data not shown). In conclusion, neuroblast migration to the OB was not generally affected by the absence of Rb.

**Figure 4:**
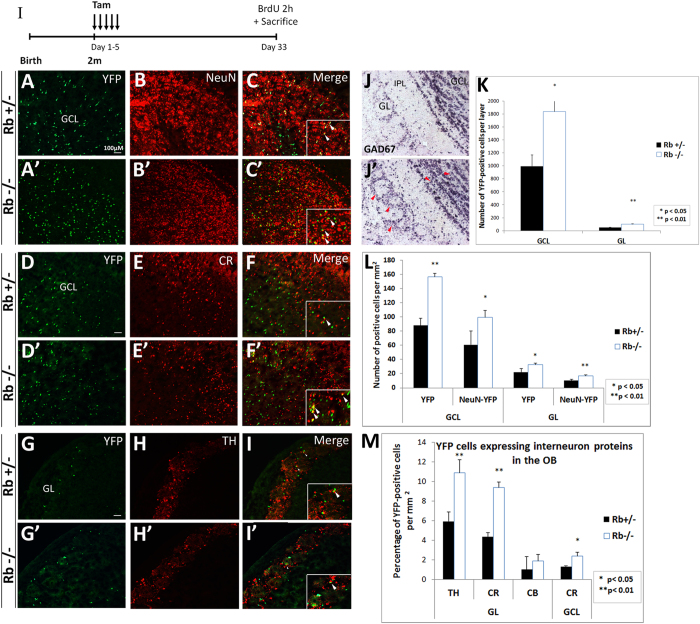
Enhanced neurogenesis in the adult OB in Rb−/− mice at 28 d post-tamoxifen treatment. (**I**) Experimental design for the temporal deletion of Rb. (**A**–**C′**) Double labeling with anti-YFP and anti-NeuN on sagittal sections in the OB. (**D**–**I′**) Co-staining of YFP+ cells with markers of GABAergic interneurons subtypes in the OB: calretinin (CR; **D**–**F′**) and tyrosine hydroxylase (TH; **G**–**I′**) (n = 3Ct and 3Mut). Insets in (**C**,**C**’,**F**,**F**’,**I**,**I**’) are higher magnifications showing double labeled cells. (**J**,**J′**) *In situ* hybridization with anti-GAD67 RNA labeled probe showing an increase in new born interneurons in the mutant OB (**J**–**J′**; red arrowheads in **J′**) (n = 3Ct and 3Mut). (**K**) Quantification revealing 1.8–2 fold increase in YFP+ cells in the OB layers in Rb−/− mice compared with controls. (**L**) Graph showing 1.5–1.8 fold increase in YFP+NeuN+ cells in the GCL and GL in the mutant OB compared with controls. (**M**) Quantification depicting increased numbers of YFP+ interneurons subtypes in the GCL (CR) and GL (CR, TH and CB) in Rb−/− vs Rb+/− mice. Error bars represent SD of measurements from n = 3 per genotype and asterisks indicate a statistically significant difference between genotypes using independent samples t-tests. Scale bar = 100 uM. IPL; internal plexiform layer, GL; glomerular layer, GCL; granule cell layer.

We next examined whether Rb−/− neuroblasts differentiate properly into mature OB neurons and thus, there is an increase in OB neurogenesis. Following their rostral migration to the OB, immature neuroblasts spread radially and terminate their differentiation as they settle in the GCL and GL of the OB approximately four weeks after their birth. They give rise to GABAergic neurons by acquiring mature morphology and establishing proper synaptic connections and up-regulate the expression of mature markers such as the nuclear protein NeuN^29^,^30^. First, we cell-sorted the green/recombinant cells found in the Rb mutant OB and confirmed that these cells were Rb-null by PCR (data not shown; see detail in supplementary methods), thus excluding the presence of a major recombination defect. Next, we checked whether Rb-null neuroblasts exited properly the cell cycle before entering the OB and did not find any ectopically proliferating YFP+ cells inside the OB in Rb−/− mice compared with controls, consistent with our previous data (Fig. 3H–H′ and data not shown). Next, we quantified the number of YFP+ cells found in the OB and detected 2-fold and 1.85-fold increase in their numbers in the GL and the GCL in Rb−/− mice compared with controls 28 d post-treatment, respectively (Fig. 4A–A′; GL: Rb+/−: 48.8 ± 7.2 vs Rb−/−: 98.22 ± 5.8, p = 0.003, and, GCL, Rb+/−: 996.1 ± 172.28 vs Rb−/−: 1839 ± 323.55, p = 0.017, mean ± S.D.). Importantly, most YFP+ cells detected in the mutant OB were NeuN-positive and thus, were mature neurons similar to those found in the Rb+/− OB (Fig. 4A–C′: GL, Rb+/−: 10.15 ± 1.6 vs Rb−/−: 16.9 ± 1.6, ratio = 1.6, p = 0.003, and, GCL, Rb+/−: 60.8 ± 19.2 vs Rb−/−: 100.5 ± 9.47, ratio = 1.64, p = 0.023, mean ± S.D.). Moreover, the ratio of [(YFP+NeuN+)/YFP+] cells inside the OB did not change suggesting the presence of a proportional increase in mature neurons with no obvious differentiation defects despite the loss of Rb (GL; Rb+/−: 45.8 ± 1.5 vs Rb−/−: 51 ± 5.5, GCL; Rb+/−: 69.1 ± 4.1 vs Rb−/−: 63.7 ± 2.3). Furthermore, we determined whether Rb-null neuroblasts differentiate into GABAergic neurons including tyrosine hydroxylase- (TH−) and calretinin- (CR−) positive interneurons, the main subtypes re-generated in the OB throughout life^31^. Compared with controls, the mutant OB displayed indeed an increase per surface area in the percentages of YFP+ cells co-expressing CR in the GCL (Fig. 4D–F′; Rb+/−: 1.3 ± 0.06 vs Rb−/−: 2.4 ± 0.3, ratio = 1.8, p = 0.037, mean ± S.D.) and TH in the GL (Fig. 4G–I′: Rb+/−: 5.9 ± 0.9 vs Rb−/−: 10.9 ± 1.3, r = 1.8, p = 0.002, mean ± S.D.) as well as CR in the GL (Fig. 4M; Rb+/−:4.4 ± 0.4 vs Rb−/−: 9.4 ± 0.6, r = 2.14, p = 0.009, mean ± S.D.). Accordingly, the expression of GAD67, one of two enzymes synthesizing GABA, was higher in the mutant OB compared with controls (Fig. 4J–J′: red arrowheads). Altogether, these data demonstrate that a large number of Rb-null progenitors migrate along the RMS and properly differentiate into adult-born interneurons similar to Rb+/− cells and survive for at least 28 d days post-Rb deletion. These findings are inconsistent with the role of Rb during embryonic development where conditional loss of Rb leads to severe migration and differentiation defects in the RMS and the OB^24^.

### Retroviral-mediated deletion of Rb in rapidly dividing SVZ progenitors does not affect neurogenesis in the OB

**Figure 5:**
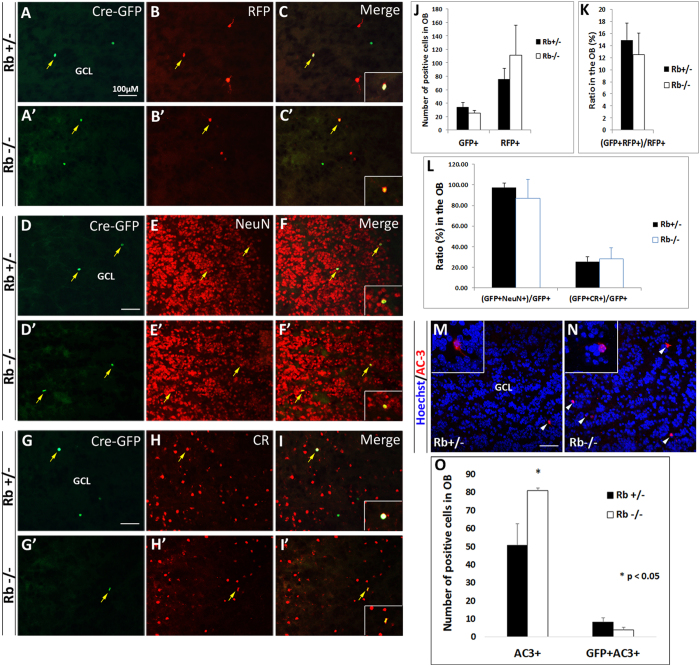
Retroviral-Cre mediated deletion of Rb does not affect neurogenesis in the adult OB. 2-month-old animals were injected with a mixture of CAG-RFP (control) and CAG-Cre/GFP retroviruses in the SVZ and sacrificed 28 d later. (**A**–**C′**) Double labeling with anti-GFP and anti-RFP on sagittal sections in the OB. (**D**–**F′**) and (**G**–**I′**) are co-labeling with (GFP, NeuN) and (GFP, CR), respectively (n = 3Ct and 3Mut). Insets in (**C**,**C′**,**F**,**F′**,**I**,**I′**) are higher magnifications showing double-labeled cells. Quantification of positive cells showed no increase in OB neurogenesis in Rb−/− mice compared with Rb+/− mice at this age (**A**–**C′**; yellow arrows, **J**,**K**). Terminal differentiation (**L**) was also similar between genotypes with the majority of GFP+ cells (87–97%) co-expressing NeuN and a subpopulation (25–28%) co-expressing calretinin (CR). The absence of enhanced neurogenesis may be explained by a dramatic increase in cell death observed in Rb-null OBs (**N**) compared with controls (**M**). Arrowheads indicate (AC3+Hoechst+) cells and insets in M and N show higher magnification images of selected cells. (**O**) Quantification of data shown in (**M**,**N**). Error bars represent SD of measurements from n = 3 per genotype and asterisks indicate a statistically significant difference between genotypes using independent samples t-tests. Scale bar = 100 uM.

To confirm the effect of Rb on adult neurogenesis in the OB, we deleted Rb in fast-dividing SVZ progenitors (type C) by retroviral-mediated Cre delivery. 8-week-old mice were stereotaxically injected in the SVZ with a mixture of CAG-Cre-GFP and CAG-RFP retroviruses, and, sacrificed 28 d later. This method allows determining the fraction of RFP-GFP double-labeled cells among the RFP-control cells; hence, represents an accurate quantitation of the influence of gene manipulation on the number of newborn OB neurons while controlling for variations that can occur from differences in injection sites and/or titers. We found that the numbers of single GFP+ cells and RFP+ cells similar between genotypes (Fig. 5A–C′) as well as the percentages of [GFP+RFP+] cells over the total number of RFP+ cells inside the OB [(Fig. 5A–C′: 14.6 ± 2.8% (11 out 75) in Rb+/− vs 12.6 ± 3.5% (14 out 111) in Rb−/−, mean ± S.D.]. This was also true for neuronal maturation as assessed by the percentage of GFP+ cells co-expressing NeuN (Fig. 5D–F′: 97.4 ± 3.8% in Rb+/− vs 86.7 ± 11.5% in Rb−/−, mean ± S.D.), and, CR in the OB, separately (Fig. 5G–I′: 25.6 ± 4.5% in Rb+/− vs 28.3 ± 10.5% in Rb−/−, mean ± S.D.). This data did not, however, reveal any increase in neurogenesis in the viral-transduced Rb−/− brains compared with controls as seen with the Nestin-CreERT2 line. To examine whether this could be due to increased apoptosis, we stained for active-caspase 3 (AC3), an apoptotic marker, and quantified cell death inside the OB. We found a significant increase in AC3+ cells but not [GFP+AC3+] cells in the absence of Rb (Fig. 5M–O). Altogether, these results confirmed that Rb is dispensable for the migration and terminal differentiation of adult-born OB neurons, yet it may be required for their survival inside the OB.

### Rb controls proliferation of adult progenitors *in vitro* without altering their multi-potential capacity

**Figure 6:**
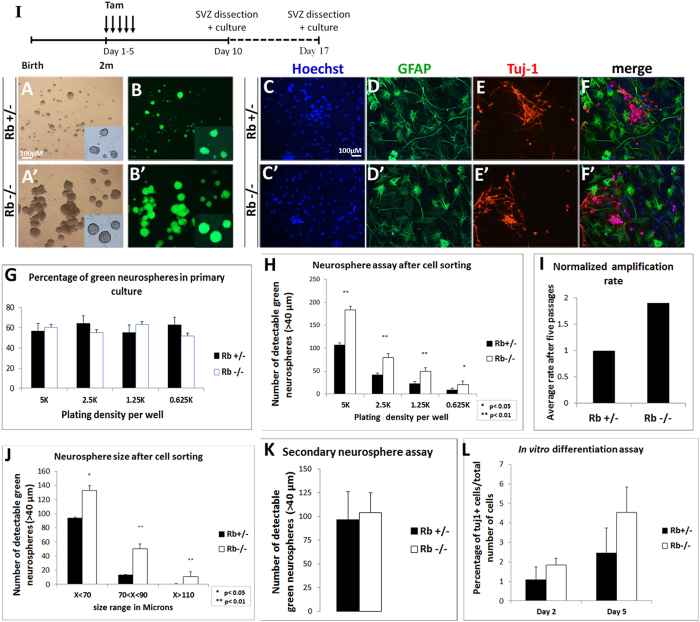
Rb controls adult aNSCs/progenitors proliferation *in vitro* without affecting their differentiation potential. (**I**) Experimental design for the neurosphere assays performed *in vitro*: 5 d and 12 d after deletion of Rb, SVZ tissues from Rb+/− and Rb−/− animals were dissected and cultured. (**A**–**A′**) Bright-field and (**B**,**B′**) fluorescent images showing the increase in the number and size of green fluorescent neurospheres generated from sorted NSCs/progenitors derived from Rb+/− vs Rb−/− cultures (n = 5Ct and 5Mut). (**C**–**F′**) Double labeling with anti-GFAP and anti-Tuj1 revealed that Rb-null progenitors differentiate into astrocytes (**D**,**D′**) and neurons (**E**,**E′**) similar to Rb+/− cells (n = 3Ct and 3Mut). (**G**) Neurosphere quantification showed that 55–60% of neurospheres generated in primary cultures are green fluorescent in Rb+/− and Rb−/− mice. (**H**) Graph showing the average number of green fluorescent neurospheres detected in five decreasing cell densities at 7 d in Rb−/− versus Rb+/− cultures, respectively. (**I**) Graph showing a 1.9 fold increase in the average amplification rate (normalized) in Rb−/− vs Rb+/− cultures after 5 passages. (**J**) Graph depicting the average size of Rb-null neurospheres detected in culture compared with controls after 7 d. The higher number of detectable spheres in the absence of Rb is due to a higher amplification rate (**I**) and reflects enhanced proliferation. (**K**) Secondary neurosphere assays were performed by dissociating single neurospheres of equal size from Rb+/− vs Rb−/− cultures and showed similar results between genotypes. (**L**) Rb-null and Rb+/− NSCs display similar neuronal differentiation rates after 2 d and 5 d in culture. Error bars represent SD of measurements from at least n = 3 per genotype and asterisks indicate a statistically significant difference between genotypes using independent samples t-tests. Scale bar = 100 uM.

Owing to the enhanced neurogenesis observed in the Rb mutant OB in Nestin-CreERT2 line, we examined how Rb controls the properties of NSCs and progenitors *in vitro.* The SVZ tissue was dissected and dissociated 5 d and 12 d following tamoxifen treatment, and neurosphere assays were performed as previously described (Fig. 6I)^3^. Passages were done every 6–7 d and dissociated cells were plated at five clonal densities (5 k, 2.5 k, 1.25 k and 0.625 k). The percentage of green fluorescent neurospheres that were detectable in primary cultures and the first passages ranged between 55–63% in Rb+/− and Rb−/− cultures indicating a relatively successful Cre recombination *in vivo* (Fig. 6G). In order to isolate the YFP+ cells, neurospheres were dissociated and cell-sorted at passage 2, then, plated as described above. All derived neurospheres were hence green fluorescent (Fig. 6A–B′). The number of detectable spheres and their ratio [Rb−/− ÷ Rb+/−] were significantly higher when comparing Rb−/− versus Rb+/− cultures on day 7 [(Fig. 6H: r = 1.71 (5 k), r = 1.9 (2.5 k), r = 2.15 (1.25 k) and r = 2.41 (0.625 K)]. Moreover, Rb-null cells showed an enhanced amplification rate (AR) that ranged between 1.5 < AR (Rb−/− ÷ Rb+/−) < 3 after five passages (Fig. 6I), and subsequently, gave rise to larger neurospheres compared with Rb+/− cells on average (Fig. 6A–B′). The above results are due to enhanced progenitor proliferation as seen *in vivo*. Next, we performed secondary neurosphere assays but did not find a significant difference in the number of detectable neurospheres between genotypes despite the consistent increase in the size of Rb-null spheres (Fig. 6K). These data suggest that Rb controls proliferation of adult progenitors but does not seem to affect the self-renewal capacity of NSCs and are consistent with our *in vivo* data. Lastly, we performed differentiation assays in order to compare the multipotency of Rb−/− vs Rb+/− NSCs/progenitors. Dissociated neurospheres were grown in monolayers to reach 60–70% confluency, then induced to differentiate for 0 d, 2 d and 5 d. Similar to control cells, Rb-null progenitors exited properly the cell cycle and differentiated into neurons and astrocytes with no significant difference in their rate of neuronal differentiation as indicated by the percentage of Tuj-1+ positive cells detected 2 d and 5 d post-differentiation (Fig. 6C–F′; %Tuj-1+ on day 5: 2.45 ± 1.24 in Rb+/− vs 4.53 ± 2.53 in Rb−/−, p = 0.295, mean ± S.D. and data not shown). Of note, we did not detect any increase in AC3+ cells 5 d post-differentiation in Rb-null culture compared with controls (data not shown). These results demonstrate that Rb controls proliferation of adult SVZ progenitors without affecting their differentiation potential *in vitro*, and are consistent with our *in vivo* findings.

### Rb is required for long-term survival of newborn neurons in the OB

**Figure 7:**
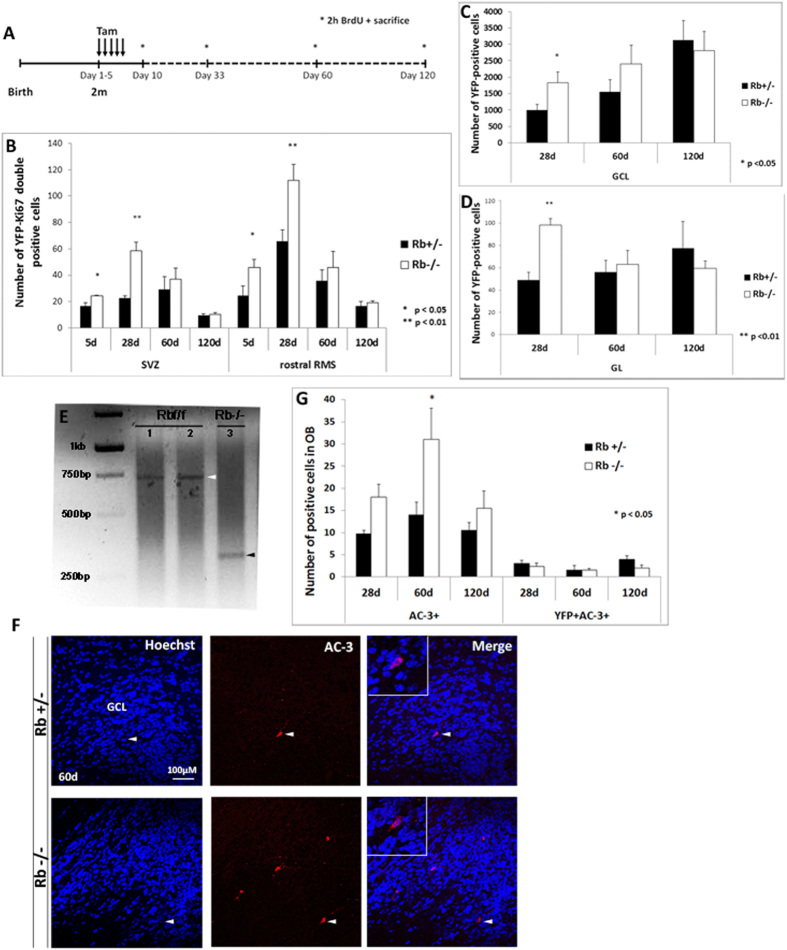
Rb is required for long-term survival of adult-born neurons in the OB. (**A**) Experimental design for the acute deletion of Rb performed in 2-month-old animals. Asterisks depict the distinct time-points of sacrifice. (**B**) Graph depicts the number of [YFP+Ki67+] cells in the SVZ and the RMS at distinct-time points in Rb−/− versus Rb+/− animals (n = 3Ct and 3Mut). Progenitor proliferation reaches a peak around 28 d in both regions but gradually decreases thereafter in both genotypes. (**C**,**D**) Graphs illustrate the number of YFP+ cells detected in the GCL (**C**) and GL (**D**) in Rb−/− vs Rb+/− mice after distinct survival periods (n = 3Ct and 3Mut). Despite the significant increase in the number of YFP+ cells found in the OBs in Rb-null brains compared with controls at 28 d, there is a gradual decrease in these cells in both layers in the absence of Rb at later stages with respect to controls. (**E**) YFP+ cells were sorted from Rb^flox/flox^ OBs at 120 d post-tamoxifen treatment (n = 6Mut) and genotyped by PCR showing that they were not Rb-null (lanes 1 and 2; white arrowhead) compared with sorted/Rb-null YFP+ cells derived in culture from 5 d dissected SVZ (lane 3; positive control, black arrowhead). (**F**) Active-caspase 3 (AC-3) staining showing a significant increase in cell death at 60 d leading to the gradual loss of Rb-null newborn neurons between 28 d and 120 d post-treatment. White arrowheads indicate double positive cells shown at higher magnification in the insets. (**G**) Quantification of data shown in (**F**) at distinct survival periods. Error bars represent SD of measurements from n = 3 per genotype and per time-point and asterisks indicate a statistically significant difference between genotypes using independent samples t-tests. Scale bar = 100 uM.

We assessed and compared the short-term versus long-term effects for the loss of Rb on the rate of adult neurogenesis in the OB. Animals received tamoxifen treatment over 5 days and were sacrificed after distinct survival periods: 5 d, 28 d, 60 d and 120 d (Fig. 7A). We found that progenitor proliferation associated with loss of Rb as assessed by the number of [YFP+Ki67+] and [YFP+BrdU+] cells peaked around 28 d in the SVZ and the RMS in Rb−/− compared with Rb+/− mice but gradually decreased thereafter between 60 d and 120 d to reach a matching level between genotypes at the latter time-point (Fig. 7B and data not shown). In parallel, the rate of OB neurogenesis as determined by the number of YFP+ cells found in the GCL gradually increased between 28 d and 120 d in control mice, however, there was no significant increase in their numbers in Rb-null brains (Fig. 7C). In addition, while the number of YFP+ cells did not significantly change in the GL in control mice over 3 months, it gradually decreased in Rb-null mice during this period to match control level at 120 d post-treatment (Fig. 7D). Sorting and genotyping of YFP+ cells found in the mutant OB at 120 d post-treatment showed that these cells were actually not Rb-null and thus derived from an incomplete recombination of the Rb^flox^ alleles (Fig. 7E, n = 6mut). Moreover, the decreased rate of neurogenesis observed in the absence of Rb overtime was directly associated with enhanced cell death that peaked at 60 d post-treatment as shown by AC-3 staining (Fig. 7F–G). These data demonstrate that loss of Rb enhances transiently neurogenesis inside the SVZ/RMS/OB and this is negatively affected by increased apoptosis, thus highlighting a requirement for Rb in the long-term survival of newborn neurons.

## Discussion

We have investigated here the role of Rb during adult OB neurogenesis and showed that Rb specifically controls proliferation of adult progenitors in the SVZ and RMS but does not affect self-renewal of NSCs and this is consistent with its role during brain development. In contrast to development, Rb-null neuroblasts undergo proper rostral migration and differentiate into distinct subtypes of OB interneurons similar to Rb+/− control cells. However, loss of Rb leads to a transient increase in SVZ/OB neurogenesis that peaks around one month later but is thereafter negatively affected by increased cell death and loss of Rb-null newborn neurons during later stages.

We have successfully deleted Rb in 75–85% of Nestin+ NSCs and progenitors found in the SVZ using an inducible Nestin-CreERT2 system (Fig. 1I–III). A large number of [Nestin+GFAP+] cells were also YFP+ at 28 d post-treatment (Fig. 2E–E′). We detected, however, a small percentage (<15%) of YFP-negative cells expressing Sox2 and GFAP, separately. These cells could be non-recombined Nestin+ stem cells/progenitors (Figs 1 and 2) and/or Sox2+ ependymal cells where minor Cre recombination was previously reported in the same Nestin-CreERT2 line^32^ and/or GFAP+ mature astrocytes. Moreover, 55–63% of neurospheres obtained in our primary cultures were green fluorescent in both genotypes (Fig. 6G), which could be partially due to an incomplete recombination e.g. the presence in the SVZ of [Nestin+YFP-] cells and/or a quiescent population of adult NSCs (qNSCs) that is [GFAP+Nestin-] and which was not targeted by our recombination system but can still give rise to colonies as described by Codega *et al.* 2014^33^. Interestingly, the latter population was shown to be molecularly distinct from the active population of [GFAP+Nestin+] NSCs that generated the majority of colonies *in vitro*^33^. Alternatively, the incomplete recombination could also result from the variable efficacy of Cre enzyme and/or the presence of non-stem cells in the adult brain that can form neurospheres^34^. Importantly, using cell sorting followed by PCR, we have verified that the green recombinant colonies of neurospheres derived from SVZ tissue in culture and the YFP+ cells found in the mutant OB at 28 d post-Rb deletion were Rb-null cells (Fig. 1II–III and data not shown). This, however, does not completely rule out the existence of a small percentage of YFP+ cells derived from an incomplete recombination e.g. Rb^flox/−^ or Rb^flox/flox^ (see discussion below).

The role of Rb in the control of adult progenitor proliferation in the SVZ and RMS is directly associated with its tumor suppressor function and consistent with its role during brain development (Figs 1,2 and 3G-H′)^22^,^24^. However, unlike during development where loss of Rb caused ectopic proliferation of cortical neuroblasts^22^ and severe migration and differentiation defects in the RMS and the OB^24^, the majority of Rb-null neuroblasts found in the adult SVZ/RMS exited properly the cell cycle and migrated rostrally to the OB where they differentiated into adult OB neurons similar to Rb+/− cells one month after their birth (Figs 3, 4, 5). This does not, however, exclude the possibility that a small number of cells underwent abnormal migration at earlier time-points. We have previously shown that the embryonic defects detected in Rb conditional Knock-out mice were attributed, at least partially, to the reduced expressions of two key differentiation genes, *Dlx1* and *Dlx2*, which are directly regulated by the Rb-E2F pathway^24^. In contrast, consistent with the absence of any major differentiation defect, the expression domains of the Dlx family of genes *Dlx1, Dlx2* and *Dlx5* as well as *GAD67* were expanded in the adult brain after loss of Rb (Figs 3E–F′ and 4J–J′ and not shown) and a large number of Rb-null ‘mature’ neurons survived at least for one month before undergoing apoptosis at later stages as described below (Figs 4, 5, 6, 7). This conclusion was strongly supported by several findings: 1) we did not find ectopically dividing YFP+ cells or significant increase in cell death inside the mutant OB compared with controls at 28 d (Fig. 7G), 2) Rb−/− mice displayed a proportional and systematic increase in cell proliferation in the brain with respect to the proliferative and mitotic indices compared to Rb+/− mice (Figs 1H,K, 2K,M and 3J), 3) we did not detect ectopic proliferation of Rb-null neuroblasts in the SVZ or RMS (Figs 1I and 3A–D′), 4) Rb-null progenitors differentiated into mature neurons and astrocytes both *in vivo* and *in vitro* (Figs 4, 5, 6C–F′). The lack of major neurogenic defects in the absence of Rb could result from compensation mechanisms mediated by other Rb family members e.g. p130 and/or p107, and suggests that the Rb pathway may be involved in distinct spatial-temporal gene regulation in adult versus embryonic neurogenesis. Future studies should address this possibility and determine also whether the increased proliferation due to loss of Rb is cell-autonomous or not and whether this could be mediated by elevated levels of FGF2 signaling as previously shown in p107- and Rb-null mice during development^35^.

Rb does not control self-renewal of aNSCs because Rb+/− NSCs and Rb-null NSCs exhibit similar rates of cell division *in vivo* and *in vitro* (Figs 1I,2L and 6K). In fact, another Rb family member, p107, was previously shown to negatively regulate the self-renewal of SVZ-NSCs in the adult brain^36^,^37^. In addition, previous studies showed that various cell cycle genes play distinct roles in the control of adult neurogenesis. For instance, loss of p53 increased the rate of division of slow- and fast-proliferating cells (type B and type C cells) and caused rapid differentiation of type C into type A cells^38^. Moreover, loss of the cyclin-dependent kinase inhibitor p27Kip1 had no effect on the number of stem cells but selectively increased the number of the transit-amplifying progenitors concomitantly with a reduction in the number of neuroblasts in the SVZ^39^. In parallel, combined loss of p53/p27Kip1 provided a proliferative advantage to SVZ populations and normal differentiation of neuroblasts^40^. Compared with other cell cycle proteins, loss of Rb enhanced progenitor proliferation and neurogenesis in the SVZ/OB with respect to the size of distinct neural populations, thus eliciting a systematic role.

The enhanced neurogenesis associated with loss of Rb was only transient and restricted to early time-points mainly 28 d post-Rb deletion in the Nestin-CreERT2 line but not in the retroviral-Cre injected brains (Figs 5A–L and 7B–D). In fact, it was opposed by increased cell death leading to loss of all Rb-null newborn neurons by 4 months post-treatment in the Nestin-CreERT2 line thus, highlighting a critical role for Rb in the long-term survival of OB neurons (Fig. 7E–G). This neuronal loss was even more pronounced in the retroviral experiments probably because the Cre-mediated recombination is more systematic when induced via viral delivery compared with tamoxifen injections (Fig. 5M–O). Of note, we did not detect an increase in [AC3+;YFP+] or [AC3+;GFP+] cells in both experimental approaches which could be due to loss of epitopes in the apoptotic cells (Figs 5O and 7G). The YFP+ population that persisted in the Rb−/− OBs, although in lower number, was not Rb-null and must have derived from a small population of [Nestin+YFP+] cells that likely underwent an incomplete recombination at earlier stages e.g. 28 d and gradually gained a selective developmental advantage compared to the YFP+ Rb-null population overtime. In one study where focal irradiation induced a 70% decrease in the number of doublecortin+ cells, a major compensatory effect on the survival of the remaining 30% of adult-born cells in the OB was observed^41^. These observations are particularly intriguing in the light of the high turnover rate of newborn neurons that normally occurs inside the OB overtime and is crucial to maintain an optimized olfaction^42^. It remains to be determined whether Rb-null neurons are functional or not, and whether they can be induced to survive for longer periods and impact olfactory function/behavior when challenged by olfactory tasks that specifically rely on adult neurogenesis such as olfactory discrimination between similar odors and short-term olfactory memory^43^.

In a recent study investigating the role of Rb in neurogenesis in the adult hippocampus using the same genetic approaches, we found that Rb-null newborn neurons were fully depleted between 15 d and 28 d, and this occurred at a faster rate compared with neurogenesis in the OB suggesting a crucial role for Rb in the survival of both adult-born granule neurons in the Hi and OB interneurons (unpublished results; Vandenbosch R, Ghanem N and Slack R, U. of Ottawa). Similarly, a study by Andrusiak *et al.* 2012 have shown that acute inactivation of Rb in post-mitotic cortical neurons induce ectopic cell-cycle proteins expression and neuronal loss both *in vitro* and *in vivo*, emphasizing a requirement for Rb for continuous cell cycle repression and survival^44^. In contrast to the above studies, it was shown that differentiated Rb−/−; p107+/−; p130−/− horizontal interneurons re-entered the cell cycle, clonally expanded, and formed metastatic retinoblastoma in mice^45^, indicating that the role of Rb in cell cycle control and survival is context-dependent and region-dependent, and there is functional crosstalk among the three pocket proteins. With respect to neurogenic sites, the environment such as the niche and the external/secreted factors might also play a significant role. Although several studies have shown significant heterogeneity in NSCs even among precursors found in the same tissue^33^,^46^,^47^,^48^,^49^, we did not detect a differential role for Rb in the regulation of distinct populations of SVZ progenitors or subtypes of OB interneurons.

In summary, we identified a novel requirement for Rb in the control of adult neurogenesis and long-term survival of newborn OB neurons which further emphasizes the role of Rb in cell cycle control and survival in the adult brain.

## Methods

### Generation of adult Rb conditional knockout mice

The Nestin-CreERT2 line was obtained from Dr. Amelia Eisch at the University of Texas Southwestern Medical Center, USA through material transfer agreement. Dr. Eisch developed the mice in collaboration with Dr. Pierre Chambon at the Institute of Genetics and Molecular and Cellular Biology (IGBMC), Strasbourg, France^25^. All animal experiments were performed in accordance with the approved guidelines of the Institutional Animal Care and Use Committee (IACUC) of the American University of Beirut, and, the University of Ottawa, which abides by the guidelines of the Canadian Council on Animal Care. Nestin is an intermediate filament protein specifically expressed in NSCs and progenitors. The Rb floxed allele has exon 19 flanked by two LoxP sites (Cre-specific sites). The yellow fluorescent protein (YFP) is separated from the ubiquitous Rosa26 promoter by a stop signal also flanked by two LoxP sites^50^. Upon administration of tamoxifen (an estrogen receptor antagonist), the Cre recombinase enzyme fused with a mutated estrogen receptor (ERT2) is trans-activated and translocates to the nucleus in cells expressing Cre under the Nestin promoter. Cre excises part of the Rb floxed allele and the stop signal from the YFP cassette, thus, inducing deletion of Rb and YFP expression in NSCs and their progeny.

### Tamoxifen treatment

30mg/ml tamoxifen solutions (Sigma T5648-5G) was dissolved in 90% sunflower seed oil (Sigma S5007) and 10% ethanol absolute (Sigma Aldrich 65533) and made daily before each injection. 180mg/kg of tamoxifen solution was administered to animals by intra-peritoneal (ip) injection for 5 consecutive days according to body weight and animals were sacrificed 5, 28, 60 and 120 days post-injections.

### Genotyping, BrdU treatment, Tissue Fixation and Cryoprotection

Detailed in Supplementary Methods.

### Immunohistochemistry, *In situ* Hybridization and Analysis of Protein Expression

Detailed in Supplementary Methods.

### Cell sorting

Detailed in Supplementary Methods.

### Cell culture

SVZ tissue was dissected from 8-week-old animals 5 d or 12 d after the last tamoxifen injection. Tissue was mechanically and enzymatically dissociated in digestion media containing [DMEM-F12 (Sigma SD8437), 0.5M EDTA (Sigma 27285), 1 μg/μl Papain (Sigma Sigma P3125)] for 30 min at 37C with gentle shaking. Next, cells were washed with 10% FBS solution [10% FBS (Sigma 14A173) in DMEM] and then re-suspended in stem cell media [DMEM-F12, 2% B27 (Unitech 17504044), 1% antibiotic/antimycotic -ABAM- (Sigma SA5955), 2 μg/ml Heparin (Sigma SH3149), 0.02 μg/ml FGF (Feldan 1D-07-017b, Invitrogen 13256-029), 0.02 μg/ml EGF (Sigma SE1257)] and plated in 10 cm petri dishes. Passages were done every 7 d and neurosphere assays were performed as follows: spheres were dissociated, counted and plated in decreasing cell densities (5 k, 2.5 k, 1.25 k and 0.625 k). At passage 2 and after dissociation, single cells were sorted using an automated cell sorter (BD FACS Aria SORP cell sorter) and plated. Neurosphere counts and size were done using an upright microscope (Olympus IX70). Single neurospheres of similar size were dissociated and plated in 96 well-plates to generate secondary neurospheres. For differentiation assays, single cells were grown in monolayer media [DMEM-F12, 1% N2 (CSMSN200-OE Gibco), 1% ABAM, 2 μg/ml Heparin, 0.02 μg/ml FGF and 0.02 μg/ml EGF] to form monolayers using 24 well-plates containing poly-ornithine (Sigma P4957) and laminin [10 ug/mL laminin (VWR-Corning 47743-734)] coated cover slides. At 60–70% cell confluency, the media was switched to differentiation media [DMEM- F12, 1% N2, 1% ABAM, 2 μg/ml Heparin, 1% FBS] and cells were differentiated for 0 d, 2 d and 5 d. Then, they were fixed for 20 minutes in 4% PFA and processed for immunostaining.

### Retroviral vectors, virus preparation and stereotactic injections

The retroviral vectors cytomegalovirus immediate early enhancer-chicken β-actin hybrid-Cre recombinase: green fluorescent protein (CAG-Cre:GFP) and CAG-red fluorescent protein (RFP) were generous gifts from F. Gage (Salk Institute, La Jolla, CA). (CAG-Cre:GFP) is also available from Addgene (plasmids #49054#). Retroviruses were generated using an all-transient transfection approach. 293T cells were transfected with a mixture containing three separate plasmids, including capsid (CMV-VsVg), viral proteins (CMV-gag/pol), and retroviral vectors using Polyethylenimine (PEI). Virus-containing supernatant was harvested twice, 48 h and 96 h after transfection, and concentrated by two rounds of ultracentrifugation. Viral titers ranged between 0.5 and 5 × 10^7^ colony-forming units (cfu) ml^−1^. For all experiments, 8-week-old males and females FVB/N Rb^flox/+^ and Rb^flox/flox^ were used. Mice were anesthetized with isoflurane and stereotactically injected with 1.5 μl of a 1:1 mixture of CAG-Cre:GFP and CAG-RFP retroviruses into the left and right SVZ. Coordinates were (in mm): −0.7 anterior/posterior ±1.2 medial/lateral from bregma and −1.9 dorsal/ventral from dura. Animals were transcardially perfused with 4% paraformaldehyde (PFA) at 28 days post injection.

### Imaging and Cell counts

Cell counts in the SVZ, RMS and OB were performed every 4^th^ sagittal section over a total thickness of ~200 μM, that is a total of 4–5 sections per level. Counts were done at three distinct levels along the lateral-medial axis of the brain. Cells expressing distinct markers were counted systematically along the whole SVZ and RMS; results from medial levels are shown in Figs 1, 2, 3 and 7. YFP-positive cells were counted exhaustively in the whole GL and GCL every 5^th^ section to cover the total OB thickness; data from medial levels with peak YFP expression are shown in Figs 4K and 7C,D. YFP cells co-expressing NeuN and distinct interneuron markers were counted in three representative regions of the GCL and the whole GL at medial level, then normalized to the surface area. Counts in Fig. 5 were done at distinct medio-lateral levels and added to obtain a representative number of RFP and/or GFP positive cells. The number and size of neurospheres in Fig. 6 were determined by analyzing all colonies in 24 well-plates. Spheres of size equal or superior to 40 μM were counted. Secondary neurospheres counts were performed in 96-well plates. Counts of Tuj-1-positive cells were conducted in 24-well plates and normalized to the number of Hoechst cells per well. Image processing and cell counts were done using Zen software (Zeiss), Image J and adobe Photoshop. All results were generated in triplicates with at least n = 3 animals per genotype and means were statistically analyzed by independent sample t-tests and Anova using the SPSS program.

## Additional Information

**How to cite this article**: Naser, R. *et al.* Role of the Retinoblastoma protein, Rb, during adult neurogenesis in the olfactory bulb. *Sci. Rep.* **6**, 20230; doi: 10.1038/srep20230 (2016).

## Supplementary Material

Supplementary Information
