# Supplementary material for: Role of the Retinoblastoma protein, Rb, during adult neurogenesis in the olfactory bulb
Source: Sci Rep. 2016 Feb 5;6:20230. doi: 10.1038/srep20230 (PMC4742828; doi:10.1038/srep20230)
Supplement: Supplementary Information [file srep20230-s1.pdf]

Role of the Retinoblastoma protein, Rb, during adult neurogenesis in the olfactory bulb

Rayan Naser[1, ł], Renaud Vandenbosch[2, ł, #], Saad Omais[1], Dayana Hayek[1], Carine Jaafar[1], Sawsan Al Lafi[1], Afaf Saliba[1], Maarouf Baghdadi[1], Larissa Skaf[1] and Noël Ghanem[1, *]

[*] **Corresponding author: ng13@aub.edu.lb**

Supplementary Methods

Genotyping Mice were weaned at post-natal day 21 (P21) and earpieces were taken for genotyping. Animals were screened using the Extract-N-Amp™ Tissue PCR Kit (Sigma-aldrich # XNAT) that allows a rapid DNA extraction and subsequent amplification of genomic DNA. The following list of primers (BioRad) were used for PCR: Rb flox primers: forward 5' GGC GTG TGC CAT CAA TG 3' and reverse 5' AAC TCA AGG GAG ACC TG 3', Nestin-Cre primers: forward 5' ATT TGC CTG CAT TAC CGG TC 3' and reverse: 5' ATC AAC GTT TTC TTT TCG G 3', ROSA26YFP primers: forward wt 5'AAA GTC GCT CTG AGT TGT TAT 3', forward mutant 5' GCG AAG AGT TTG TCC TCA ACC 3', and reverse 5' GGA GCG GGA GAA ATG GAT ATG 3'.

BrdU treatment To assess proliferation, a single IP injection of bromodeoxyuridine (BrdU) was administered to all animals 2 hours before sacrifice according to body weight: 10 mg/ml BrdU (Sigma B5002-250MG) solution was dissolved in 0.9% NaCl and 0.007N NaOH. For birth-dating studies, 5 BrdU injections were given 3 days after tamoxifen treatment: one injection given every 2h over a 10h period. Animals were sacrificed and analyzed 28d later.

Tissue fixation and cryoprotection Prior to sacrifice, mice were euthanized with 1.5µl/g ketamine and 0.25µl/g Xylazine and subjected to cardiac perfusion with 20-25ml cold phosphate buffer solution 1x

(PBS) followed by 15-20ml of 4% cold paraformaldehyde (PFA) (ACROS). Brains were then dissected and post-fixed in 4% PFA overnight. Next day, brains were rinsed with 1x PBS and cryoprotected using 20% sucrose in 1x PBS for 2 days followed by 30% sucrose for 4-5 days. Finally, brains were embedded in Tissue-Tek O.C.T. (SAKURA 4583) and frozen on dry ice using cold isopentane (-35°C) (Sigma Aldrich M32631). 8-10μm thick sagittal sections were generated using a cryostat (Leica, CM1850) and mounted on SuperFrost adhesion slides (Fisher scientific and Thermo Scientific) and stored at -80°C.

Immunohistochemistry Frozen sections were warmed at room temperature -RT- for 30 minutes, then blocked for at least 1 hour in blocking solution [1% BSA (amresco 0332-25G), 0.3% Triton X, 5% goat or donkey serum in 0.1M PBS]. Primary antibodies were applied to sections overnight at RT. The next day, following 3x washes in 1x PBS for 10 minutes each, sections were incubated in secondary antibodies (1:400) along with Hoechst stain (1:100) for 2 hours. Finally, the sections were washed 3 times in 1x PBS for 10 minutes each and mounted using 1xPBS/Glycerol (3:1). The following primary antibodies were used: chicken anti-YFP (1:1000; Abcam ab13970), rabbit anti-YFP (1:3000; Chemicon AB6556), rabbit anti-RFP (1:5000, Abcam ab62341), rabbit anti-Tyrosine Hydroxylase (TH) (1:750; Chemicon), rabbit anti-Calretinin (CR) (1:4000; Swant), rabbit anti-Calbindin (CB) (1:500; Chemicon AB1778), mouse anti-BrdU (1:50; BD Pharmagon 347580), rat anti-BrdU (1:500; Accurate OBT0030), goat anti-double cortin (DCX) (1:500; Santa Cruz sc8066), mouse anti-Ki67 (1:500; BD Pharmigen 550609), rabbit anti-Ki67 (1:500, Cell Marque), rabbit anti-Tuj-1 (1:5000, Covance, PRB-435P), mouse anti-NeuN (1:750; Millipore MAB377), guinea pig anti-Dlx2 (1:2000, precious gift from gift from K. Yoshikawa, Institute for Protein Research, Osaka University, Suita, Osaka, Japan), goat anti-Sox2 (1:150, Santa Cruz), goat anti-GFAP (1:100, Santa Cruz, sc-6170), rabbit anti-active caspase 3 (1:500, Cell Signaling 9664) and rabbit anti-GFAP (1:2000, Dako Z0334). The secondary antibodies (Alexa Fluor, Molecular Probes, Invitrogen, and, Jackson Immunoresearch) used were: goat and chicken anti-rabbit 488, goat anti-chicken 488, goat and chicken anti-mouse 596, goat and chicken anti-rabbit 596, goat anti-rat 596, donkey anti-

goat 596, chicken anti-rabbit 647, chicken anti-goat Cy3, chicken anti-goat 647 and chicken anti-guinea pig 647.

BrdU labeling slides were incubated for 45 seconds in acetone, then washed for 10 min in 1xPBS and incubated in 1N HCl for 20 min at 37°C. Slides were then neutralized in 0.1M Sodium Borate (Na2B4O7; pH=8.5) (Fisher scientific S-249) for 10 min and finally washed with 1x PBS. Antibodies were then applied as described earlier.

Antigen retrieval Antigen retrieval was performed before staining for nuclear proteins including NeuN, Ki67, Sox2 and Dlx2. Slides were treated with fresh 10mM sodium citrate (Fisher scientific BP327-500), pH=6 for 15 min at 95C or, alternatively, incubated in target retrieval solution (Dako, S1700) in similar conditions. Following antigen retrieval, slides were washed in 1x PBS and YFP signal was amplified using TSA (Tyramide Signal Amplification) kit (PerkinElmer- ABC kit Fluorescein SAT701001EA).

In situ hybridization *In situ* hybridization on frozen tissue sections using digoxigenin (DIG) labeled RNA probes was performed according to the procedures previously described [1]. Hybridized probes were detected with an AP-conjugated anti-digoxigenin Fab fragment antibody (1:2000, Roche) and visualized with the NBT/BCIP substrate system. Antisense riboprobes for *Dlx2* [2], *Dlx5* and *GAD67* (generous gifts from J. L. Rubenstein, University of California at San Francisco, San Francisco, CA), were prepared from plasmids.

Analysis of protein expression *Protein* lysates were prepared from cultured green neurospheres (after cell sorting) in Universal Lysis Buffer, as previously described [3]. Electrophoresis was performed using 8% Tris-Chloride gels, and proteins were transferred to polyvinylidene difluoride membranes (Bio-Rad Laboratories, Hercules, CA), and detected using antibodies to total RB (BD-Pharmingen, San Diego, CA, Cat#554136, 1:250) and GAPDH (Santa Cruz, Sc25778, 1:1000). The secondary antibodies used are: goat anti-mouse (Sc-2005, Santa Cruz 1:2500) and goat anti-rabbit (Sc2004, Santa Cruz, 1:2500).

Cell sorting the olfactory bulbs from 5-6 animals carrying the same genotype were dissected and mechanically broken up into small pieces. Tissues were then pooled together and treated with digestion media for 30' at 37C as described above. After digestion, tissues were gently triturated twice and re-suspended in DMEM/F12 with 10% FBS to inactivate papain. Then, tissues were triturated again and re-suspended in 100%Percoll-PBS buffer solution, for a final concentration of 22% vol./vol. Percoll. Following 15min centrifugation at 500g, dissociated cells were filtered using 40uM filter to obtain single cells suspension in DMEM-F12 without phenol red. Cells were subsequently processed for cell sorting (BD FACS Aria SORP cell sorter).

References

1. Wallace VA, Raff MC. A role for Sonic hedgehog in axon-to-astrocyte signalling in the rodent optic nerve. **Development**. 1999;126:2901-2909.
2. Porteus MH, Bulfone A, Ciaranello RD et al. Isolation and characterization of a novel cDNA clone encoding a homeodomain that is developmentally regulated in the ventral forebrain. **Neuron**. 1991;7:221-229.
3. Zalzali H, Harajly M, Abdul-Latif L et al. Temporally distinct roles for tumor suppressor pathways in cell cycle arrest and cellular senescence in Cyclin D1-driven tumor. **Molecular cancer**. 2012;11:28.
